# Review of Hematology-Oncology Emergencies for Internal Medicine Residents

**DOI:** 10.7759/cureus.33563

**Published:** 2023-01-09

**Authors:** Bohdan Baralo, Nithya Ramesh, Sohiel Deshpande, Bhanusowymya C Buragamadagu, Aliza Khanam, Mahati Paravathaneni, Sana Mulla, Verushka Bedi, Vihitha Thota, Raisa Baralo, Akhil Jain, Eugene Choi, Rajesh Thirumaran

**Affiliations:** 1 Internal Medicine, Mercy Catholic Medical Center, Darby, USA; 2 Clinical Pharmacy and Clinical Pharmacology, National Prigov Memorial Medical University, Vinnytsia, UKR; 3 Hematology/Oncology, Mercy Catholic Medical Center, Darby, USA

**Keywords:** internal medicine residency, educational practicies, critical care, oncology emergencies, hematology emergencies

## Abstract

The prevalence of cancer continues to grow globally every year. With therapeutic advances over the recent decades, the prevalence of individuals living with cancer continues to increase. Internal medicine residents can see patients admitted to the hospital for cancer-related emergencies. Early identification and appropriate management of these emergencies have been shown to improve mortality and morbidity. In this article, we aim to review the recent updates in the management of commonly encountered oncologic emergencies in the practice of internal medicine residents. This review will cover spinal cord compression, superior vena cava syndrome, tumor lysis syndrome, hypercalcemia, pericardial tamponade, hypoglycemia, hyponatremia, bowel obstruction, increased intracranial pressure, leukostasis, hyperviscosity syndrome, neutropenic fever, and hypersensitivity reactions.

## Introduction and background

In 2020, an estimated 1,806,509 new cases of cancer were diagnosed in the United States, with 606,520 anticipated deaths. Cancer mortality rates have decreased since the early 1990s, with 1.8% per year among men and 1.4% per year among women. As such, the number of survivors has increased. As of January 2019, there were an estimated 16.9 million cancer survivors, an increase of 22.2 million by 2030 [1]. Some oncologic emergencies are insidious and take months to develop, whereas others manifest over hours, causing significant mortality and morbidity [2]. Prompt identification of these emergencies and timely intervention can improve survival and the quality of life [3]. Internal medicine residents should be aware of the following hematology-oncology emergencies, which they are likely to encounter during their training and later on, in independent practice: spinal cord compressions, superior vena cava (SVC) syndrome, tumor lysis syndrome (TLS), hypercalcemia, pericardial tamponade, hypoglycemia, syndrome of inappropriate antidiuretic hormone (SIADH) secretion, bowel obstruction, increased intracranial pressure (ICP), leukostasis, hyperviscosity syndrome, neutropenic fever, and hypersensitivity reactions. In this article, we provide a review of these emergencies.

## Review

Spinal cord compression

Metastatic spinal cord compression is a catastrophic oncologic emergency that occurs in 10%-20% of patients with cancer. It prompts early diagnosis and treatment to preserve the quality of life, including the prevention of functional loss. Reportedly, up to 21% of patients experience spinal cord compression due to metastasis as a diagnostic event [4,5]. Prostate, lung, breast, renal cell cancer, multiple myeloma, and Hodgkin lymphoma are some that can lead to spinal cord compression [5]. Varied clinical presentations pose a diagnostic challenge. New onset back pain, with or without focal neurological deficits, is some of the common symptoms. Patients without neurological deficits should be evaluated within 24-48h of symptoms onset, whereas those with neurological deficits should be evaluated promptly.  Magnetic resonance imaging (MRI) and computed tomography (CT) myelograms are the mainstays in diagnosis [6]. ​ Treatment consists of steroids, radiation therapy (RT), chemotherapy, and surgery.​ A standard dose of steroids (16 mg dexamethasone IV bolus followed by 4-6 mg Q4-6 H) followed by rapid tapering after initiating RT is preferred early in management. Spine instability neoplastic scores (Table 1) is a valuable tool that can be used universally to determine the need for surgical stabilization. A score ≥ 7 in these patients warrants immediate neurosurgical evaluation and possible intervention. Management often requires a multimodal approach after discussing the goals of care with the patient [5-7].

**Table 1:** Spine instability neoplastic score Spine Instability Neoplastic Score was first described by Fischer et al. and used to determine patients who might benefit from surgical stabilization. [5] C - cervical vertebra, T - thoracic vertebra, L - lumbar vertebrae, S- sacral vertebra

Element	Score
Location	
Junctional (occiput-C2, T7-T2, T11-L1m L5-S1)	3
Mobile spine (C3-C6, L2-L4)	2
Semirigid spine (T3-T10)	1
Rigid spine (S2-S5)	0
Pain with recumbency and /or movement of the spine	
Yes	3
Occasional, but not mechanical	1
No	0
Bone lesion	
Lytic	2
Mixed (lytic and blastic)	1
Blastic	0
Radiographic spinal alignment	
Subluxation or translation present	4
De novo deformity	2
Normal alignment	0
Vertebral body collapse	
>50%	3
<50%	2
No collapse, with>50% involved	1
None	0
Involvement of posterolateral spinal elements (facet, pedicle, or costovertebral joint fracture or replacement with a tumor)	
Bilateral	3
Unilateral	1
None of the above	0
Total score	
Stable	0-6
Indeterminate	7-12
Unstable	13-18

SVC syndrome 

SVC syndrome encompasses symptoms of jugular vein obstruction at various levels. The most common etiologies include lung carcinoma, lymphoma, germ cell tumors, thymic malignancies, mesothelioma, and metastases [2]. Non-malignant causes, such as mediastinal fibrosis, aneurysms, iatrogenic catheters, implantable cardioverter-defibrillators, and pacemakers, contribute to approximately 40% of the cases [7]. Presenting symptoms can include swelling of the face and neck, distension of the neck veins, cough, dyspnea, stridor, hoarseness, facial plethora, upper body edema, headache, altered mental state, stupor, coma, vision changes, syncope, nausea, and dysphagia. SVC syndrome can be life-threatening in the event of airway compromise. 

Although selective venography is the gold standard test, chest CT with contrast is preferred, given its availability and with a sensitivity and specificity of 96% and 92%, respectively [2,3,7]. Vein dilation, a pathognomonic sign of SVC, can take up to two weeks to develop [3]. The treatment depends on the etiology and severity of the symptoms [8]. Airway protection and endovascular stenting remain a mainstay in emergencies. Surgery, palliative or active chemotherapy, and radiotherapy are other options in nonemergent cases. Treatment of SVC caused by thrombosis involves thrombolysis and/or device removal.

Tumor lysis syndrome

TLS results from the release of contents from cancer cells, resulting in hyperkalemia, hyperphosphatemia, hypocalcemia, and hyperuricemia. ​TLS is most common in patients with hematologic malignancies and solid tumors (small cell lung cancer, germ cell tumors, inflammatory breast cancer, and melanoma) [7,8]. ​It can be provoked by chemotherapy, RT, surgery, or ablation, occurring soon after treatment or delayed by several weeks.

The presence of at least two components of the Cairo-Bishop criteria is diagnostic. Notably, laboratory (uric acid ≥8 mg/dL, potassium ≥6 mEq/L, phosphorus ≥4.5 mg/dL, calcium ≤7 mg/dL, or change in any value listed >25% from baseline) and clinical (creatinine ≥1.5 above baseline, or cardiac arrhythmia/sudden death, or seizure) [8,9].

The expert risk panel developed a risk stratification tool dividing all patients at low (<1% chance of TLS), medium (1%-5%), and high risk (>5%) based on age, histology, the extent of disease, type of cytotoxic agents used, and pretreatment renal function [8,9].  The medium-risk patients are recommended to have adequate hydration and allopurinol for the primary prevention of TLS, while high-risk patients are recommended Rasburicase in addition [8,10,11].

Management includes aggressive intravenous hydration while monitoring urine output. Alkalization of urine is not routinely recommended; however, if used, it should be discontinued if hyperphosphatemia develops. Rasburicase is the preferred agent. It is contraindicated in pregnancy and Glocose-6-Phosphate Dehydrogenase (G6PD) deficiency. Hyperphosphatemia is managed with phosphorus binders.​ Hyperkalemia is treated with temporizing measures (insulin, dextrose, calcium gluconate, and albuterol) and potassium binders (patiromer, sodium-zirconium cyclosilicate, and sodium polystyrene sulfonate). Electrolyte imbalance, renal failure, volume overload, and metabolic acidosis refractory to conservative treatment may prompt dialysis.  If present, symptomatic hypocalcemia (tetany, carpopedal spasm, seizures, or prolonged QTc) should be treated using intravenous calcium gluconate [10,11].

Hypercalcemia

The incidence of hypercalcemia in malignancy has been reported to be between 10%-33% [12]. Hypercalcemia is most commonly caused by humoral mechanisms (80%) and osteolytic metastases (20%) [2]. Humoral mechanisms include the secretion of parathyroid hormone-related protein (PTHrP) or extrarenal 1,25 dihydroxy vitamin D (calcitriol). PTHrP can be secreted by squamous cell carcinoma, breast cancer, renal carcinoma, prostate cancer, melanoma, and neuroendocrine tumors. It binds to parathyroid hormone (PTH) receptors in bones and kidneys, enhancing bone resorption and calcium retention [13]. Increased production of calcitriol occurs almost exclusively in Hodgkin and non-Hodgkin lymphomas due to the expression of the 1-alpha-hydroxylase enzyme in macrophages. Rarely, ectopic PTH production by ovarian/lung cancers and some neuroendocrine tumors can occur. At the same time, osteolytic bone metastases increase calcium levels in multiple myeloma, leukemia, and metastatic breast carcinoma.   

Hypercalcemia of malignancy is severe due to very high calcium (serum calcium >14 mg/dL, >3.5 mmol/L), occurring over weeks to months. Symptoms are usually nonspecific, including anorexia, nausea, vomiting, apathy, constipation, polydipsia, polyuria, abdominal pain, and muscle weakness. Severe cases may result in neuropsychiatric manifestations like malaise and lassitude, progression to confusion, and coma. QT interval shortening and dysrhythmias may also occur [12]. 

Initial workup includes ionized calcium, albumin, PTH, PTHrP, 25-hydroxyvitamin D, and calcitriol levels. In humoral hypercalcemia of malignancy, PTHrP is elevated, with low to low-normal PTH, phosphorus, and calcitriol. Metastatic causes result in low PTH and calcitriol, with undetectable PTHrP. Lymphoma with increased calcitriol levels also demonstrated low 25-hydroxyvitamin D and undetectable PTHrP [12].   

Treatment includes hydration using intravenous isotonic saline (initial 1-2 L bolus, followed by continuous fluids). After euvolemia is achieved, intravenous Lasix is initiated 1-2 times/day to facilitate calcium excretion, especially in patients with oliguric renal failure, congestive heart failure, or symptomatic volume overload following aggressive hydration. Intramuscular or subcutaneous calcitonin is then administered. The onset of action occurs within 4-6 hours, and the effect lasts up to 48-72 h. Calcitonin acts by inhibiting osteoclasts and enhancing urine calcium excretion. However, the standard of care is an intravenous bisphosphonate (pamidronate/zoledronate), with an onset of action within 2-4 days until which calcitonin exerts its effect. Zoledronate is ~1,000 times more potent than pamidronate because of the superior inhibition of osteoclastic enzymes. Some adverse effects include fever, bone pain during infusion, osteonecrosis of the maxilla and mandible (<1% of patients), uveitis, and nephrotoxicity. Bisphosphonates should be renally dosed and generally avoided in patients with GFR <30 mL/min. Glucocorticoids are reserved for calcitriol-mediated hypercalcemia because they inhibit 1-alpha hydroxylase-mediated calcitriol production. Denosumab (receptor activator of nuclear factor kappa-Β ligand (RANKL) antibody that inhibits osteoclast maturation and activation) is used to manage bisphosphonate-resistant malignant hypercalcemia [14]. Dialysis is reserved for refractory cases. The prognosis is poor, with a median survival time of 35 days [2].

Pericardial tamponade 

Pericardial tamponade is fluid accumulation between the visceral and parietal pericardium, leading to hemodynamic collapse.​ Classic symptoms include Beck's triad of hypotension, muffled heart sounds, and increased jugular venous distention. On physical examination, pulsus paradoxus can be detected (systolic blood pressure decreased by ≥ 10 mmHg on inspiration).

Pericardial mesothelioma, lung cancer, and lymphoma​ are the most common causes of malignant pericardial effusion [15]. It can also develop as a complication of RT. Diagnostic tests that detect pericardial effusion include chest radiography (bottle-shaped heart), electrocardiogram, point-of-care ultrasonography, or CT. Every patient with signs, symptoms, or additional workup suggestive of pericardial effusion should undergo the bedside echocardiogram to evaluate for ultrasound signs of tamponade: diastolic right ventricular collapse, and systolic right atrial collapse, plethotic, non-collapsible inferior vena cava, and dondgraphic pulsus paradosux [16].

Hemodynamically unstable patients require emergency pericardiocentesis.​ Pericardiocentesis under echocardiographic guidance is recommended in nonemergent cases. The recurrence rate is decreased in pericardiocentesis with extended catheter drainage or balloon pericardiotomy.  Patients should be treated for primary malignancy (chemotherapy vs. RT)​.  Alternative treatment options for recurrent effusions include pericardial window, pericardiectomy, catheter placement, balloon pericardiotomy, and pericardial sclerotherapy [3,15].

Hypoglycemia 

Hypoglycemia is a blood glucose level < 72 or 4 mmol/L. It causes various autonomic symptoms, such as paresthesia, sweating, palpitations, weakness, mydriasis, tremor, hunger, tachycardia, and anxiety due to adrenergic counter-regulation, and neuroglycopenic symptoms such as irritability, dizziness, blurring of vision, confusion, altered mental status, transient focal neurological deficits, seizures, and coma. The most common malignant causes of hypoglycemia include excessive insulin production (insulinomas), pancreatic beta-cell dysfunction (nesidioblastosis), and hypersecretion of catecholamines (pheochromocytoma) and insulin-like growth factor (IGF) (IGF-2 by sarcomas, gastrointestinal stromal tumors, solitary fibrous tumors, and IGF 3 by lung cancer). Altered metabolism due to parenchymal destruction in hepatocellular carcinomas and increased consumption in rapidly multiplying neoplasms (Burkitt lymphoma, small cell lung cancer) are other causes [3,17]. Diagnosis involves history, clinical signs and symptoms, and physical examination. Whipple's triad includes low blood glucose levels, symptoms of hypoglycemia, and relief of symptoms with the restoration of normal blood glucose levels.

When there is high clinical suspicion without symptoms or hypoglycemia, patients should undergo a 72-hour fasting test. Insulin, proinsulin, C-peptide, and β-hydroxybutyrate levels should be measured during hypoglycemic episodes. IGF-1,2 is measured if other results are non-diagnostic.

CT, transabdominal ultrasound (US), and MRI are used to diagnose insulinomas [18]. Endoscopic US and fine-needle aspiration may be required if localization is not possible noninvasively. A selective arterial calcium stimulation test with hepatic venous sampling can be used to distinguish insulinomas from nesidioblastosis and islet cell hypertrophy [19]. Acute hypoglycemia is managed with 25g of 50% dextrose intravenously. 0.5-1.0 mg of subcutaneous, intramuscular, or intranasal glucagon can be administered until intravenous access is established.

Surgical tumor removal is the optimal approach to avoid future hypoglycemic episodes. Medical management of insulinoma includes diazoxide, octreotide, or lanreotide. Diazoxide decreases insulin secretion but can cause marked edema and hirsutism [20]. Diazoxide and somatostatin analogs do not play a role in non-islet cell tumors. Non-selective beta-blockers are sometimes used to reduce the sympathetic symptoms of hypoglycemia.  The mainstem of treatment for unresectable tumors is chemotherapy or RT (to decrease hormonal activity) and glucocorticoids, with frequent carbohydrate intake to control hypoglycemic symptoms. For hypoglycemia, refractory to high-dose glucocorticoids, long-term intravenous glucagon infusion, or recombinant human growth hormone (prevents IGF binding to insulin receptors) is an option. However, growth hormones can also increase IGF-1 levels and tumor growth [21].

Hyponatremia

Hyponatremia is defined as a serum sodium level <135 mEq/L. The incidence of hyponatremia is the highest with small-cell lung cancer [22]. Per observational studies, hyponatremia is associated with extended hospital stays and increased mortality among cancer patients [23].

Causes include SIADH secretion, brain metastasis, chemotherapy (cisplatin, cyclophosphamide, ifosfamide, vinca alkaloids, imatinib, and methotrexate), and adverse effects, including nausea and vomiting. Some medications, including opioids, antidepressants (tricyclic antidepressants, selective serotonin reuptake inhibitors, and monoamine oxidase inhibitors), non-steroid anti-inflammatory drugs, antipsychotics (haloperidol), and antiepileptics (carbamazepine, oxcarbazepine, and valproic acid) are other cause of hyponatremia [22,24]. 

The patients’ volume should be assessed​ and corrected. Serum and urine osmolality, urine sodium, plasma uric acid, blood urea nitrogen (BUN), thyroid-stimulating hormone (TSH), and morning cortisol levels should be obtained to rule out hypothyroidism and hypercortisolism. Serum osmolality <275 mOsm/kg, urine osmolality >100 mOsm/kg, urine sodium >40 mmol/L, plasma uric acid <4 mg/dL, and BUN <10 mg/dL suggest SIADH [25].  Fluid intake of asymptomatic and euvolemic patients should be restricted to 500-1,000 mL/day. Symptomatic patients with mild-to-moderate hyponatremia should receive vaptans (colvaptan/tolvaptan)​. Severe symptomatic hyponatremia (defined as the presence of neurological symptoms) should be treated with a hypertonic saline​ solution. It is recommended that sodium levels be corrected no faster than 0.5 mEq/L/H to avoid central pontine myelinolysis [25].

Bowel obstruction

Bowel obstruction is a frequent complication in advanced malignancies. It may be caused by intrinsic or extrinsic compression by the tumor (colonic, pancreatic, ovarian, or stomach), ascitic fluid (peritoneal carcinomatosis), postsurgical and radiotherapy adhesions, or due to opioids [26].

Symptoms include abdominal pain, distention, constipation, constipation, and vomiting. Bowel movements are initially hyperactive but eventually cease.  Warning signs include tachycardia, hypotension, electrolyte abnormalities, fever, chills, leukocytosis, and lactic acidosis. Signs of peritonitis suggest microbial translocation into the abdominal cavity, bowel wall necrosis, and/or perforation ​[27].

The initial management steps are to keep the patient nil per oral, place a nasogastric tube (NGT) for decompression, and use intravenous fluids to cover gastrointestinal loss, antiemetics, and correction of electrolyte and acid-base disorders. Surgical evaluation is required immediately, followed by abdominal CT.​ Prokinetic agents may be beneficial in partial obstruction, and some studies have demonstrated successful corticosteroid application for short-term relief from malignant obstruction.  Emergent surgery is indicated if bowel necrosis/perforation is suspected [27]. Uncomplicated obstruction can be managed by surgical resection of the tumor with anastomosis, proximal diversion with an ostomy, or bypass. Self-expanding metal stents can be placed if surgical intervention is not possible [28]. Some trials have demonstrated the effectiveness of somatostatin analog, lanreotide, as an antiemetic in inoperable obstruction caused by peritoneal carcinomatosis, allowing for the removal of the NGT to optimize patient comfort [29].

Increased ICP 

About 20%-40% of cancer patients are at risk of developing brain metastases [30]. Melanoma, lung, renal, breast, and colon cancers are the common primary tumors to metastasize. Increased ICP is usually caused by the mass effect of primary and metastatic brain tumors, vasogenic edema from increased vascular permeability, or inflammatory reaction secondary to RT [30,31].

Common symptoms are new-onset/worsening headaches, nausea, vomiting, focal neurological deficits, and/or seizures. ​Cushing's triad indicates increased ICP and possible impending herniation and is composed of hypertension, bradycardia, and bradypnea.  Contrast-enhanced MRI is the gold standard for diagnosis. Noncontrast CT is used to rule out hemorrhage/hydrocephalus urgently. Watershed areas and grey-white matter junctions are common locations of metastasis.

Elevated ICP is managed with dexamethasone 10-24 mg IV bolus followed by 4 mg every 6-8 h. ​Mannitol, an osmotic diuretic, can be used temporarily in patients without dehydration. Intubation with hyperventilation is reserved for patients with rapidly declining mentation as a bridging therapy to emergent craniotomy [32].

Definitive treatment like stereotactic radiation therapy, surgery, and systemic or intrathecal chemotherapy (lymphomas, germ cell tumors, and small cell carcinoma) is often necessary to reduce tumor burden.​ Whole-brain radiation is reserved for patients with poor prognoses due to adverse neurological effects [31].

Leukostasis 

Leukostasis is associated with leukocytosis and is often seen in acute myelocytic leukemia (AML) (5%-13%) or acute lymphocytic leukemia (ALL) (10%-30%) [3,33]. The most common subtypes are M3, M4, and M5 of AML, ALL with 11q23 mutation, or Philadelphia chromosome [3,33]. Children, especially infants, are the most affected, with up to 40% mortality if not treated timely [3]. The pathophysiology involves increased blood viscosity, complement-mediated granulocyte aggregation, and the interaction between leukemic blasts and endothelium. The severity of symptoms depends on the cell size (more pronounced in AML) and response to chemotactic cytokines but is not related to the number of blasts [3,34].

Symptoms include fever, exertional dyspnea, respiratory failure, neurological manifestations like confusion, headache, dizziness, blurry vision, visual field defects, focal symptoms from a brain hemorrhage, and early death.  Other manifestations include myocardial infarction, renal vein thrombosis, disseminated intravascular coagulation (DIC), papilledema, retinal hemorrhage, and limb ischemia [3]. The incidence of DIC is 30%-40% in AML (mostly M3 subtype) and 15%-20% in ALL [33]. WBC count is the most important prognostic factor, and a count > 50,000 indicates poor prognosis and early mortality [3]. CT brain is required in suspected cases to rule out brain hemorrhage, and chest radiography may reveal multifocal infiltrates. Rapid cytoreduction with induction chemotherapy (decreases WBC count in 24 hours) is the preferred treatment. TLS is a common adverse effect and is managed as mentioned above. Hydroxyurea can be used to decrease leukocytes in asymptomatic patients if immediate induction chemotherapy is unavailable. A dose of 1-2 g q6h (50-100 mg/kg/day) of hydroxyurea can reduce the WBC count by 50%-80% in 24 to 48h [3,32]. Leukapheresis can be done in symptomatic patients or WBC counts >100,000 (>200,000 in ALL) but is contraindicated in acute promyelocytic leukemia (APML or M3 subtype) as it worsens thrombocytopenia and may cause DIC [3]. In APML, all-trans-retinoic acid is started to help the blast cells' maturation and cytoreduction [33]. Low RBC counts often counterbalance the encountered leukocytosis. Hence, packed RBCs should be avoided unless the hemoglobin is <7-8 g/dL or if the patient is symptomatic [3,33]. Cranial or pulmonary irradiation has rarely been performed as a temporizing measure in patients with severe neurological or respiratory symptoms [3,34].

Hyperviscosity syndrome 

Hyperviscosity can be observed in disorders of the serum and whole blood components. The size and shape of the proteins in serum determine their contribution to viscosity. IgM protein has a large axial length and increases viscosity more than IgG and IgA immunoglobulins. Common causes include Waldenstrom macroglobulinemia (WM), multiple myeloma, rituximab IgM flare, type I and II cryoglobulinemia, polycythemia vera, HbSS, CLL, chronic myeloid leukemia (CML), and ALL. Increased immunoglobulins causing hyperviscosity are also seen in human immunodeficiency virus (HIV) infection, Sjogren's syndrome, high titers of rheumatoid factor, and IVIg infusions [35-37].

The classic presentation of hyperviscosity is seen with the triad of visual changes (retinal hemorrhages, papilledema), neurological abnormalities (seizure, ataxia, cerebral hemorrhage), and mucosal bleeding (gingival bleeding, epistaxis). Involvement of the cardiovascular system, such as high-output cardiac failure with paraproteins >8 g/L, may also be present. Symptoms occur especially with a paraprotein level of 5-8 g/L [35].

**Figure 1:**
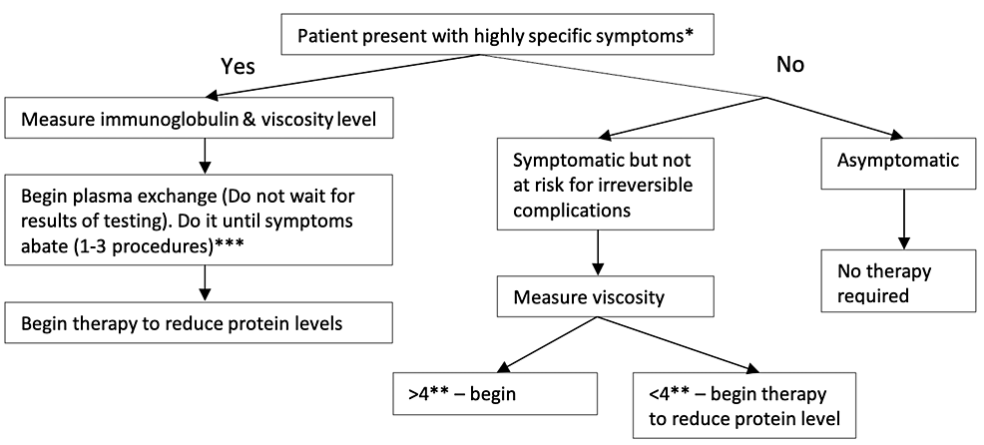
Hyperviscosity syndrome management *Highly specific symptoms: mucosal hemorrhage (bilateral epistaxis, gingival, gastrointestinal, retinal), visual disturbance (bilateral retinal hemorrhage or thrombosis, papilledema, blurring), neurologic (somnolence or coma, cerebral hemorrhage,, seizure, ataxia), high output heart failure ** Measured in relative plasma viscosity, centipose (cp: relative to water: normal range, 1.4-1.8 cp) *** Duration of treatment per Guidelines in the Use of Therapeutic Apheresis in Clinical Practice [39]

Treatment depends on the presence of symptoms and viscosity levels, and apheresis is considered the gold standard (Figure 1) [35]. Although not required in symptomatic patients, plasma viscosity measurement helps in guiding treatment. Plasma exchange can effectively reduce 30%-50% viscosity in one session. Daily or every other day, plasma exchange is repeated until symptoms abate (1-3 procedures). Typically, one session is enough to reduce plasma viscosity to levels where mucosal hemorrhage will not occur [35,38,39]. Phlebotomy may be used if plasmapheresis is unavailable.

For asymptomatic patients, systemic chemotherapy can be considered. For example, bortezomib-based or ibrutinib-rituximab-based treatment effectively decreases protein levels in patients with WM [38,40].

Neutropenic fever 

Neutropenic fever is defined as temperature (>100.4 F/38 C) in a patient with decreased absolute neutrophil count (ANC). Neutropenia is defined as ANC<1000 (ANC<500 = severe; ANC<100 = profound). Neutropenia in malignancy predisposes to severe infections. It is commonly caused by the cytotoxic effect of chemotherapy (usually peaking at 5-10 days of treatment), impaired hematopoiesis, or due to metastatic infiltration of the bone marrow. ​Commonly implicated chemotherapeutic agents are anthracyclines, topoisomerase inhibitors, vinorelbine, cyclophosphamide, ifosfamide,​ and taxanes [3,41].

The initial workup includes a complete blood count, two sets of blood cultures, cultures of the suspected source of infection (urine, sputum, etc.), basic metabolic panel, liver function tests, and lactate levels [3]. Several tools have been developed to estimate the necessity of hospital admission for patients with neutropenic fever, such as the Multinational Association of Supportive Care in Cancer (MASCC) index (Table 2), clinical index of stable febrile neutropenia (CISNE) prognostic class (Table 3), and Talcott grouping. Talcott classification contains four groups: first group is inpatient, second is outpatient with active comorbidity requiring admission, third is patient without comorbidity and uncontrolled cancer, and fourth is outpatient with well-controlled cancer and no comorbidities. Patients with MASCC index over 21, Talcott group 4, and CISNE point less than 2 meet the outpatient management criteria. They can be treated empirically with a combination of fluoroquinolones and amoxicillin/clavulanate. Clindamycin may be used in patients allergic to penicillin. Further evaluation is advised if the fever recurs or persists after 48 hours [42,43].

**Table 2:** MASCC score Multinational Association of Supportive Care in Cancer (MASCC) risk index factors and their individual weights. Used in clinical practice to determine the necessity for inpatient hospitalization in patients with neutropenic fever [42,43].

Characteristic	Score
Burden of febrile neutropenia with no or mild symptoms	5
No hypotension (systolic blood pressure > 90 mmHg)	5
No chronic obstructive pulmonary disease	4
Solid tumor or hematologic malignancy with no previous fungal infection	4
No dehydration requiring parenteral fluids	3
Burden of febrile neutropenia with moderate symptoms	3
Outpatient status	3
Age <6o years	2

**Table 3:** CISNE score Clinical Index of Stable Febrile Neutropenia (CISNE) factors and score. Used in clinical practice to determine the necessity for inpatient hospitalization in patients with neutropenic fever [42,43].

Explanatory variable	Score
Eastern Cooperative Oncology Group performance status >/= 2	2
Stress-induced hyperglycemia	2
Chronic Obstructive Pulmonary Disease (COPD)	1
Chronic cardiovascular disease	1
Mucositis of grade >/= 2	1
Monocytes < 200/ microliter	1

Inpatient treatment is recommended for those who fulfill the criteria or fail outpatient treatment. Ideally, treatment should be started within one hour of presentation with intravenous broad-spectrum antibiotics with pseudomonal coverage (cefepime, piperacillin-tazobactam, and meropenem), even if risk stratification has not been completed [41]. Delays in initiating antibiotics have been correlated with an increase in the length of hospital stay [42]. Vancomycin can be added for suspected catheter-related infections, skin and soft tissue infections, pneumonia, and hemodynamic instability. Antibiotics are modified once culture results are available.

Hypersensitivity reactions 

Antineoplastic therapy (commonly taxanes) is the third leading cause of fatal drug-induced anaphylaxis in the United States [44]. Hypersensitivity reactions can occur via two different pathways: immune-mediated (IgE pathway leading to an allergic reaction, anaphylaxis), and non-immune (pseudo-allergic reactions due to cytokine release syndrome).  Organ-specific symptoms include mucocutaneous (pruritus, rash, desquamation, hives), respiratory (dyspnea, bronchospasm, and acute laryngopharyngeal dysesthesia usually caused by oxaliplatin), circulatory (hypo-or hypertension, shock), fever, and chills. Patient-related risk factors include older age, female sex, preexisting comorbidities, history of allergic reactions, and severe atopic disease. Anaphylactoid response to paclitaxel/docetaxel, due to direct effects on immune cells (mast cells), is often observed with first-time infusion [45,46].

In severe cases, the infusion must be stopped. Intramuscular epinephrine (0.3-0.5 mg, can be repeated at 3-5 min) should be administered with supportive measures of oxygen supplementation, intravenous fluids, steroids, and antihistamines. ​In mild cases, the infusion should be temporarily discontinued while administering diphenhydramine and steroids. Infusion can be restarted at a slow rate, with close patient monitoring if symptoms resolve. 

Recent studies have shown the merits of risk-stratifying patients who develop hypersensitivity reactions after the first drug exposure. Patients with mild or delayed reactions can be desensitized or rechallenged with the treatment agent. It is beneficial in patients with promising responses to chemotherapy or who have limited treatment options [47,48].  

## Conclusions

In this review article, we covered the most common hematology and oncology emergencies that internal medicine residents can encounter during their practice. Unfortunately, the onset of cancer complications can be insidious and well ahead of symptoms caused by the tumor itself. Thus, proper diagnosis and treatment can be delayed, potentially leading to death or significant morbidity. This review covers several oncology and hematology emergencies and will be helpful to early internal medicine trainees to get familiar with diagnostic and treatment modalities.

## References

[1] (2022). National Cancer Institute. Cancer statistics. https://www.cancer.gov/about-cancer/understanding/statistics.

[2] Higdon ML, Atkinson CJ, Lawrence KV (2018). Oncologic emergencies: recognition and initial management. Am Fam Physician.

[3] Lewis MA, Hendrickson AW, Moynihan TJ (2011). Oncologic emergencies: pathophysiology, presentation, diagnosis, and treatment. CA Cancer J Clin.

[4] Savage P, Sharkey R, Kua T (2014). Malignant spinal cord compression: NICE guidance, improvements and challenges. QJM.

[5] Fisher CG, DiPaola CP, Ryken TC (2010). A novel classification system for spinal instability in neoplastic disease: an evidence-based approach and expert consensus from the Spine Oncology Study Group. Spine (Phila Pa 1976).

[6] Arana E, Kovacs FM, Royuela A, Asenjo B, Pérez-Ramírez Ú, Zamora J (2016). Spine Instability Neoplastic Score: agreement across different medical and surgical specialties. Spine J.

[7] Annemans L, Moeremans K, Lamotte M (2003). Incidence, medical resource utilisation and costs of hyperuricemia and tumour lysis syndrome in patients with acute leukaemia and non-Hodgkin's lymphoma in four European countries. Leuk Lymphoma.

[8] Cairo MS, Coiffier B, Reiter A, Younes A (2010). Recommendations for the evaluation of risk and prophylaxis of tumour lysis syndrome (TLS) in adults and children with malignant diseases: an expert TLS panel consensus. Br J Haematol.

[9] Cairo MS, Bishop M (2004). Tumour lysis syndrome: new therapeutic strategies and classification. Br J Haematol.

[10] Pui CH, Mahmoud HH, Wiley JM (2001). Recombinant urate oxidase for the prophylaxis or treatment of hyperuricemia in patients with leukemia or lymphoma. J Clin Oncol.

[11] Howard SC, Jones DP, Pui CH (2011). The tumor lysis syndrome. N Engl J Med.

[12] Sternlicht H, Glezerman IG (2015). Hypercalcemia of malignancy and new treatment options. Ther Clin Risk Manag.

[13] Yeung SCJ, Pollock RE, Weichselbaum RR (2003). Holland-Frei Cancer Medicine. 6th edition.

[14] Hu MI, Glezerman IG, Leboulleux S (2014). Denosumab for treatment of hypercalcemia of malignancy. J Clin Endocrinol Metab.

[15] Maisch B, Ristic A, Pankuweit S (2010). Evaluation and management of pericardial effusion in patients with neoplastic disease. Prog Cardiovasc Dis.

[16] Alerhand S, Adrian RJ, Long B, Avila J (2022). Pericardial tamponade: a comprehensive emergency medicine and echocardiography review. Am J Emerg Med.

[17] Mathur A, Gorden P, Libutti SK (2009). Insulinoma. Surg Clin North Am.

[18] Noone TC, Hosey J, Firat Z, Semelka RC (2005). Imaging and localization of islet-cell tumours of the pancreas on CT and MRI. Best Pract Res Clin Endocrinol Metab.

[19] Thompson SM, Vella A, Thompson GB, Rumilla KM, Service FJ, Grant CS, Andrews JC (2015). Selective arterial calcium stimulation with hepatic venous sampling differentiates insulinoma from nesidioblastosis. J Clin Endocrinol Metab.

[20] Gill GV, Rauf O, MacFarlane IA (1997). Diazoxide treatment for insulinoma: a national UK survey. Postgrad Med J.

[21] Bodnar TW, Acevedo MJ, Pietropaolo M (2014). Management of non-islet-cell tumor hypoglycemia: a clinical review. J Clin Endocrinol Metab.

[22] Castillo JJ, Glezerman IG, Boklage SH, Chiodo J 3rd, Tidwell BA, Lamerato LE, Schulman KL (2016). The occurrence of hyponatremia and its importance as a prognostic factor in a cross-section of cancer patients. BMC Cancer.

[23] Doshi SM, Shah P, Lei X, Lahoti A, Salahudeen AK (2012). Hyponatremia in hospitalized cancer patients and its impact on clinical outcomes. Am J Kidney Dis.

[24] Liamis G, Milionis H, Elisaf M (2008). A review of drug-induced hyponatremia. Am J Kidney Dis.

[25] Castillo JJ, Vincent M, Justice E (2012). Diagnosis and management of hyponatremia in cancer patients. Oncologist.

[26] Prenen K, Prenen H (2015). Oncological emergencies associated with gastrointestinal tumors. Ann Gastroenterol.

[27] Long B, Robertson J, Koyfman A (2019). Emergency medicine evaluation and management of small bowel obstruction: evidence-based recommendations. J Emerg Med.

[28] Franke AJ, Iqbal A, Starr JS, Nair RM, George TJ Jr (2017). Management of malignant bowel obstruction associated with GI cancers. J Oncol Pract.

[29] Mariani P, Blumberg J, Landau A (2012). Symptomatic treatment with lanreotide microparticles in inoperable bowel obstruction resulting from peritoneal carcinomatosis: a randomized, double-blind, placebo-controlled phase III study. J Clin Oncol.

[30] Loeffler JS, Sawaya R., H H, S. DeVita VT Jr, Rosenberg SA (1997). Cancer: Principles and Practice of Oncology.

[31] Brown PD, Ahluwalia MS, Khan OH, Asher AL, Wefel JS, Gondi V (2018). Whole-brain radiotherapy for brain metastases: evolution or revolution?. J Clin Oncol.

[32] Lin AL, Avila EK (2017). Neurologic emergencies in the patients with cancer. J Intensive Care Med.

[33] Majhail NS, Lichtin AE (2004). Acute leukemia with a very high leukocyte count: confronting a medical emergency. Cleve Clin J Med.

[34] Porcu P, Cripe LD, Ng EW, Bhatia S, Danielson CM, Orazi A, McCarthy LJ (2000). Hyperleukocytic leukemias and leukostasis: a review of pathophysiology, clinical presentation and management. Leuk Lymphoma.

[35] Gertz MA (2018). Acute hyperviscosity: syndromes and management. Blood.

[36] Dimopoulos MA, Tedeschi A, Trotman J (2018). Phase 3 trial of ibrutinib plus rituximab in Waldenström’s macroglobulinemia. N Engl J Med.

[37] Fahey JL, Barth BF, Solomon A (1965). Serum hyperviscosity syndrome. JAMA.

[38] Siddarath B, Agrawal D, Deshpande S (2020). Epidemiology and outcomes of hospitalizations due to Waldenstrom macroglobulinemia: a national perspective. Blood.

[39] Padmanabhan A, Connelly-Smith L, Aqui N (2019). Guidelines on the use of therapeutic apheresis in clinical practice - evidence-based approach from the writing committee of the American Society for Apheresis: the eighth special issue. J Clin Apher.

[40] Ballestri M, Ferrari F, Magistroni R (2007). Plasma exchange in acute and chronic hyperviscosity syndrome: a rheological approach and guidelines study. Ann Ist Super Sanita.

[41] Taplitz RA, Kennedy EB, Bow EJ (2018). Outpatient management of fever and neutropenia in adults treated for malignancy: American Society of Clinical Oncology and Infectious Diseases Society of America clinical practice guideline update. J Clin Oncol.

[42] Perron T, Emara M, Ahmed S (2014). Time to antibiotics and outcomes in cancer patients with febrile neutropenia. BMC Health Serv Res.

[43] Mohindra R, Mathew R, Yadav S, Aggarwal P (2020). CISNE versus MASCC: identifying low risk febrile neutropenic patients. Am J Emerg Med.

[44] Jerschow E, Lin RY, Scaperotti MM, McGinn AP (2014). Fatal anaphylaxis in the United States, 1999-2010: temporal patterns and demographic associations. J Allergy Clin Immunol.

[45] Hong D, Sloane DE (2019). Hypersensitivity to monoclonal antibodies used for cancer and inflammatory or connective tissue diseases. Ann Allergy Asthma Immunol.

[46] Jhaveri KD, Wanchoo R, Sakhiya V, Ross DW, Fishbane S (2017). Adverse renal effects of novel molecular oncologic targeted therapies: a narrative review. Kidney Int Rep.

[47] Bonamichi-Santos R, Castells M (2018). Diagnoses and management of drug hypersensitivity and anaphylaxis in cancer and chronic inflammatory diseases: reactions to taxanes and monoclonal antibodies. Clin Rev Allergy Immunol.

[48] Picard M, Pur L, Caiado J (2016). Risk stratification and skin testing to guide re-exposure in taxane-induced hypersensitivity reactions. J Allergy Clin Immunol.

